# Using a theory of change for evaluation: has the FAIMER international faculty development program improved the field of health professions education?

**DOI:** 10.15694/mep.2019.000050.1

**Published:** 2019-03-12

**Authors:** Shiyao Yuan, Snigdha Mukherjee, Rashmi Vyas, William Burdick

**Affiliations:** 1 Foundation for Advancement of International Medical Education and Research

**Keywords:** International Faculty Development Program, Health Professions Education, Theory of Change, Program Evaluation

## Abstract

This article was migrated. The article was marked as recommended.

Program theories have not been extensively used in evaluating faculty development programs in medical education. Ample evidence shows that a well-formulated program theory plays a pivotal role in program implementation and evaluation. Program theory links activities and expected outcomes using a logical process showing how they lead to long-term goals. It also develops appropriate metrics or indicators for assessing if those outcomes and activities really occurred. In this study, FAIMER’s theory of change was adopted as a framework for evaluation. Survey data from FAIMER Fellows was used to assess the effectiveness of FAIMER’s faculty development program in meeting the goal of improving health professions education. We used structural equation modeling to examine the association among outcomes mapped out in our theory of change and their association with improving field of health professions education. The study results indicated that FAIMER’s faculty development program appeared to have positively influenced advancement of multiple facets of health professions education as envisaged in our theory of change. Using a theoretical framework for evaluating a program helped us identify the specific areas of outcomes that need to be strengthened for program improvement as well as provided us with a data-driven evaluation framework to measure program progress.

## Introduction

Program theory, or theory of change, is an important tool for the implementation of faculty development programs, yet it has not been extensively used in program evaluation in medical education. There is ample evidence showing that a well-formulated program theory plays a pivotal role in providing a framework for evaluation ( Grol *et al.*, 2007; Foy *et al.*, 2011; French *et al.*, 2012). Program theory maps out activities or interventions that will lead to the expected outcomes identified as preconditions for achieving long-term goals. Program theory also helps develop appropriate metrics or indicators for assessing if those outcomes really occurred. In a recent review of faculty development programs, Steinert and colleagues (2016) suggested that future evaluation research should use program theory as the foundation for the assessment of program impact as it offers an opportunity to evaluate hypotheses implicit in a concatenated series of outcomes. The need for more effective use of theory in program evaluation is increasingly pressing ( Davidoff *et al.*, 2015). In this study, we used program theory to assess an international faculty development program for health professions educators.

In 2001, Foundation for Advancement of International Medical Education and Research (FAIMER) initiated a two-year faculty development program - FAIMER fellowship program - designed for international, mid-career health professions educators who have demonstrated the potential to improve health professions education at their institutions in resource-limited countries ( Norcini, Burdick and Morahan, 2005; William P. Burdick, Morahan and Norcini, 2006). As part of the application process, each candidate (a participant of the fellowship program) proposes an education innovation project that is endorsed by their home institution and then implements it when they return. The education innovation project, which serves as the focal point for application of learning, is implemented during the course of fellowship program. Many projects continue to be developed and expanded after Fellows complete the program. Each year of the program consists of a short-term (1-3 weeks) residential component and a long-term (11 months) distance learning component. Distance learning is organized through an email listserv with Fellows’ active engagement in online learning discussion groups. As intentional community building, this facilitates the development of a network of educators to help Fellows stay connected during and after the fellowship program.

**Figure 1.**
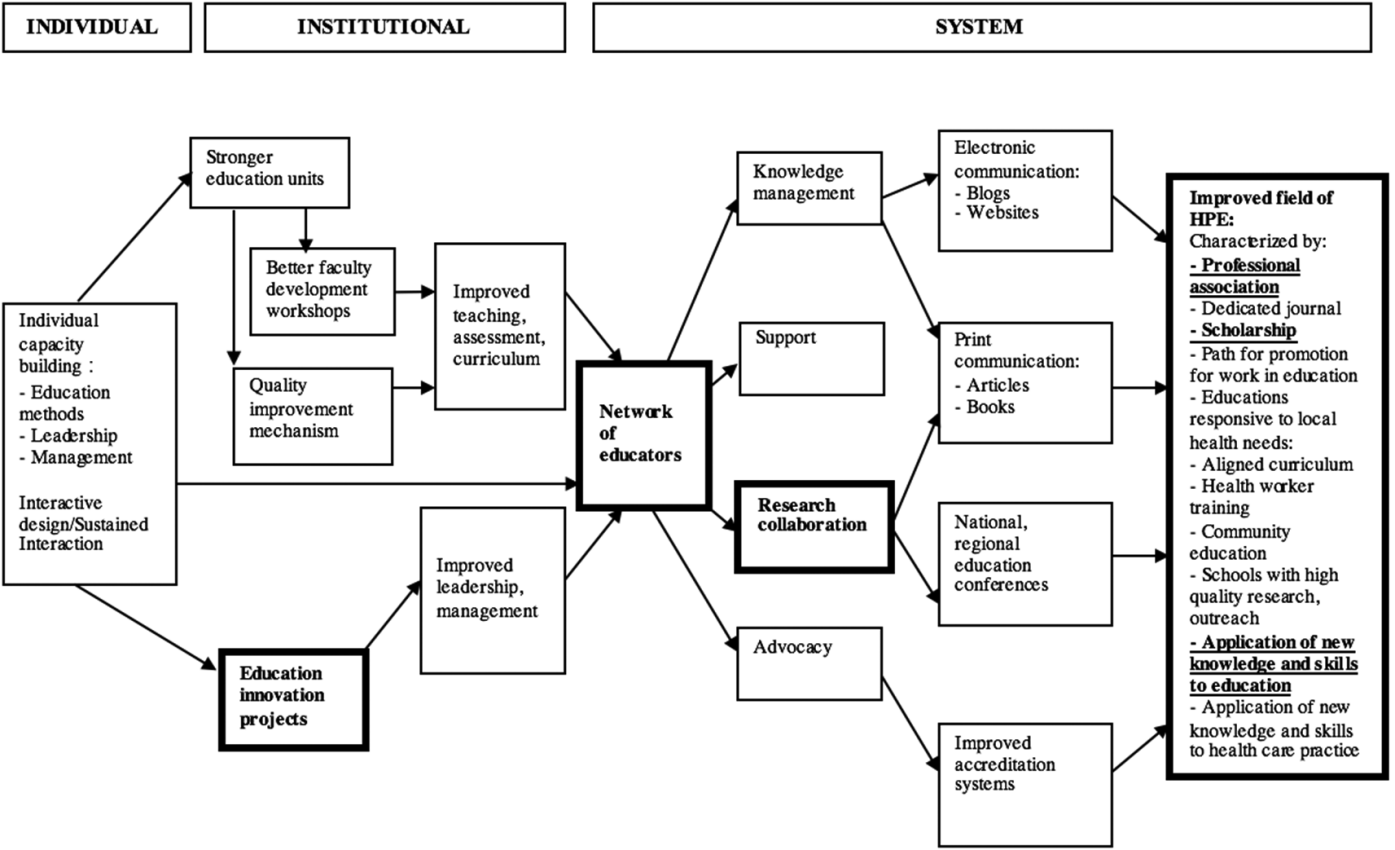
FAIMER Theory of Change, first published in *A model for linkage between health professions education and health: FAIMER international faculty development initiatives* ( Burdick W et al. 2011), reproduced with permission from Taylor & Francis.

FAIMER’s theory of change was created as the program was being developed ( William Burdick *et al.*, 2011), with the goal of improving the field of health professions education (see **Figure 1**). Evidence of field improvement includes development of a health professions education association, application of new knowledge and skills acquired through the fellowship program, and education scholarship such as publications and conference presentations. Development of projects ( Gusic *et al.*, 2010; Steinert *et al.*, 2016), enhanced professional networks or community of practice ( Simpson *et al.*, 2004; O’Keefe *et al.*, 2009; Steinert *et al.*, 2016) and research collaboration ( O’Sullivan and Irby, 2011) have been described as important elements of faculty development programs and are also key outcomes in FAIMER’s theory of change. These interrelated outcomes depict the anticipated ripple effect of FAIMER’s fellowship programs.

Demonstrating a relationship between outcomes in a theory of change is one way to provide evidence for the validity of a program theory. In many cases, outcomes in theory of change are latent constructs that cannot be directly observed or measured, and structural equation modeling (SEM) method is often used in this situation to estimate the relationship among theoretical latent constructs ( Kline, 2005; Schreiber *et al.*, 2006; Violato and Hecker, 2007). SEM has been applied to theory validation extensively in a variety of research problems in social sciences ( Kandemir, Yaprak and Cavusgil, 2006; Klassen and Chiu, 2010; Heimler, Rosenberg and Morote, 2012; Kusurkar *et al.*, 2013; Moreira *et al.*, 2016) and to a limited degree in medical education research ( Humphris, 2002; Collin, 2004; Chiu *et al.*, 2016). In this study, we utilized SEM to evaluate FAIMER fellowship programs by exploring the association among four intermediate outcomes or latent constructs. We are specifically seeking answers to the following questions:

1. Does implementation of education innovation projects positively affect development of a network of educators?

2. Does development of a network of educators positively affect research collaboration among Fellows and faculty?

3. Do research collaboration, education innovation projects, and/or network of educators have a positive effect on the field of health professions education?

## Methods

### Participants

Participants in this study included Fellows who either completed a FAIMER fellowship program (alumni Fellow) or just completed the first year of a FAIMER fellowship program (current Fellow) during the data collection period. Fellows from all fellowship programs were included in the study.

### Measurement

Fellows were asked to answer an online survey which was designed to longitudinally track their professional activities and growth, as well as their ongoing involvement with the FAIMER community. Survey items were designed to measure the four latent constructs in FAIMER’s theory of change. All survey items were designed as binary “yes/no” checklists and the selected four latent constructs in theory of change were measured by corresponding survey items based on theory. The constructs of development of a network of educators (“ *Network of Educators”*), implementation of education innovation projects (“ *Education Innovation Projects”*), “ *Research Collaboration*,” and positive effect on the field of health professions education (“ *Improved field of HPE”*) are multi-dimensional and therefore reflected by several underlying sub-constructs measured by survey items.

#### Education Innovation Projects

Items related to *Education Innovation Projects* were grouped into two sub-constructs labelled *Project Status* and *Change(s) by Projects.* *Project Status* (9-item) denoted the current status of Fellow’s FAIMER project. A list of possible project status was provided (e.g. “my project has been incorporated into the curriculum” or “my project has been expanded to address additional objectives that were not originally part of the project”) and Fellows checked the relevant statements that applied to their project status. *Change(s) by Projects* (17-item) referred to changes that have occurred to the school and community/region as a direct or indirect result of Fellow’s project. Fellows checked statements that applied to their own project (e.g. “there is more faculty interest in the quality of teaching” or “student performance on knowledge and outcome measures has improved”).

#### Network of Educators

The construct *Network of Educators* was measured by sub-constructs *Community Involvement* and *Community Gain.* *Community Involvement* (14-item) indicated Fellows’ involvement with the FAIMER community during the past one year. Examples of items included “meeting with FAIMER Fellow(s) at a professional meeting” and “participation on the listserv by reading posts”. *Community Gain* (13-item) indicated skills and knowledge Fellows perceived they have gained through involvement with the FAIMER community during the past one year, such as “new ideas about HPE, deeper knowledge about specific content areas in HPE” and “leadership advice”.

#### Research Collaboration

Four survey items related to research collaboration were aggregated as a single construct *Research Collaboration* that revealed whether Fellows have collaborated with another Fellow or faculty on research opportunities during the past one year such as publications, educational materials, conference organizing/presenting, or grants.

#### Improved field of HPE

The construct *Improved field of HPE* was measured by six sub-constructs, grouped in three categories: Scholarship, including *Conference Organizing/Presenting (3-item)* and *HPE Publications (2-item),* recorded whether Fellows had any scholarship accomplishments in the past one year. Items of interest include “Did you publish any HPE indexed articles last year?” and “Did you organize or present at a national conference?” *HPE Organization Association* (2-item), *HPE Unit Association* (2-item), and *HPE Journal Association* (2-item), measured Fellow’s professional association related to health professions education during the past year. Examples of items include “Are you a member of any HPE organization?”, “Were you involved in the creation of the HPE unit?”, and “Have you been on the editorial board of an HPE journal?” Application of new knowledge and skills to education, denoted by *Knowledge/skills Application (14-item),* identified areas of application of knowledge and/or skills Fellows had gained from FAIMER, such as “curriculum development or revision” and “workshop development or facilitation”.

### Data Collection

The online survey was emailed to 1,049 Fellows (current Fellows n=303, alumni Fellows n=746) from whom we had documented consent. SurveyMonkey, an online survey software application, was used for survey administration and data collection. Responses from current Fellows were collected at the beginning of their second year of fellowship in 2016 (for 2015 class Fellows) and in 2017 (for 2016 class Fellows). Responses from alumni Fellows (2001 to 2013 class) were collected from September 2016 to December 2016.

### Analysis

De-identified responses were exported and then merged with the Fellows’ demographic data including gender, age, fellowship program, and year of enrollment. Univariate frequency distribution of the item responses was examined to understand the data pattern. Since survey item responses were binary, we used tetrachoric correlations to examine association between items measuring the same latent construct. To reduce model noise and avoid model overfitting, item responses with low frequency (< 100) and that were poorly correlated with other items measuring the same latent construct or sub-construct (coefficient <0.3) were dropped from the final dataset. For example, for *Community Involvement*, only 28 (4.4%) selected “DAFFRI (Director and Faculty of FAIMER Regional Institutes) participant, and 65 (10.3%) selected “FAIMER Institute Faculty”, and these two items were poorly correlated with other items, thus were dropped from the final dataset. Moreover, items conceptually defined as a combination of other indicators were also excluded to avoid linear dependency. For example, for *Change by Project*, besides the checklist of items that give examples of changes by project, there is also an item “I am not aware of any changes in my school or community/region that have occurred as a result of my project”. This item can be conceptually defined as a combination of a non-selection of other indicators. Therefore, we dropped this item from the final dataset. After data cleaning, the final dataset includes a total of 41 survey items (see **Table 1**).

**Table 1.** Confirmatory Factor Analysis components related to survey questions

Constructs/sub-constructs (Abbreviation)	Question items
Community Involvement (COMM_INV)	CI1 - Met with FAIMER Fellow(s) at a professional meeting CI2 - Participation on the listserv by reading postings CI3 - Participation on the listserv by contributing postings CI4 - Communication with individual FAIMER Fellows (not including listserv communication) CI5 - Communication with individual FAIMER Faculty (not including listserv communication)
Community Gain (COM_GAIN)	CG1 - New ideas about health professions education CG2 - Deeper knowledge about specific content areas in health professions education CG3 - Leadership advice CG4 - Research collaboration CG5 - Sharing resources
Knowledge/Skills Application (KS_APPLY)	KS1 - Curriculum development/revision KS2 - Training course or workshop development/facilitation KS3 - Educational advisor or consultant, either formal or informal KS4 - Student assessment KS5 - Health Professions/Medical Education Unit creation, expansion, or improvement KS6 - Faculty development program creation, expansion, or improvement
Current Status of Project (PJ_STATUS)	PS1 - My project has been incorporated into the curriculum PS2 - My project has been incorporated as an institutional policy or procedure PS3 - My project (or aspects of it) is being replicated in another course/module/year at my institution PS4 - My project is being applied in another setting in my country PS5 - My project is being applied in another setting in another country PS6 - My project has been expanded to address additional objectives that were not originally part of the project
Changes to Institution or Community/Region as a Direct or Indirect result? of Project (PJ_CHANGE)	PC1 - There is more faculty interest in the quality of teaching PC2 - The quality of teaching has improved PC3 - Curriculum is better aligned with community health needs PC4 - There is more intradepartmental collaboration in education (including education research, teaching, etc.)
Publication (PUB)	PU1 - Did you publish any HPE indexed articles last year? PU2 - Did you create any educational materials that are publically available during the past one year (e.g. available in a repository, publication, online venue that is available to the public?)
Conference organization or presentation (CON)	CO1 - Organized or presented at a national conference CO2 - Organized or presented at a regional conference CO3 - Organized or presented a an international conference
Research Collaboration (COLLAB)	RC1 - Did you collaborate with another FAIMER Fellow on any publications during the past one year? RC2 - Did you collaborate with another FAIMER Fellow on any educational materials during the past one year? RC3 - During the past one year, did you collaborate with another FAIMER Fellow on any HPE conferences (This means collaboration via co-presenting or co-organizing)? RC4 - Did you collaborate with FAIMER Fellow or FAIMER Faculty member on any grant?
HPE Organization Association (HPE_ORG)	HO1 - Have you been a member of an HPE organization? HO2 - Have you been a leader of an HPE organization?
HPE Unit Association (HPE_UNIT)	HU1 - Were you involved in the creation of the HPE unit? HU2 - Are you a member of the HPE unit?
HPE Journal Association (HPE_JOUR)	HJ1 - Have you been a peer reviewer of an HPE journal during the past year? HJ2 - Have you been on the editorial board of an HPE journal during the past one year?

SEM analysis was implemented in two stages: measurement model and structural model. In the measurement model, confirmatory factor analysis was used to determine structural validity of the survey measurement through examining factor loadings of survey items on the corresponding latent construct and sub-construct. For multi-dimensional constructs, i.e. *Education Innovation Project*, *Network of Educators*, and *Improved field of HPE*, second-order confirmatory factor analysis was also used to examine the factor loadings of sub-constructs on the corresponding constructs. We also calculated Cronbach’s alpha for survey items measuring each construct and sub-construct to assess internal consistency. In the structural model, path analysis was used to examine the association among the four selected constructs in our theory of change. SEM was conducted using Mplus version 7.4. We used weighted least square mean - variance (WLSMV) estimation to accommodate the categorical nature of response data, since WLSMV does not assume normally distributed variables and provides the best option for modeling categorical or ordered data ( Brown, 2006). The following indices were referenced to access model fit: chi square ( *X ^2^*), comparative fit index (CFI), Tucker Lewis index (TLI), and root mean square error of approximation (RMSEA). We considered the model demonstrated a good fit if CFI and TLI exceeded 0.9 and RMSEA was less than 0.05 ( Kline, 2005; Violato and Hecker, 2007). Modification indices were also referenced to examine whether there were significant paths that were not in the model but supported by empirical data, and paths with index value greater than 30 were added to the model.

## Results/Analysis

### Response Rate and Participant Demographics

The study successfully enrolled 632 (current Fellows n = 283, alumni Fellows n = 349) out of a total of 1049 (current Fellows n=303, alumni Fellows n=746) for a total response rate of 60.2%. Of the 632 enrolled Fellows, 581 fully completed the questionnaire and 51 partially completed the questionnaire. There was no significant difference in gender between respondents and non-respondents (60% vs 59%, *X ^2^* =.154, p > 0.05). Respondents were on average 4.6 years younger than non-respondents (46.2 ± 8.5 vs 50.8 ± 8.9, t = 8.0, p < 0.01).

### Model fit

The final model fit ( **Table 2**) had the following characteristics:n = 632, *df* = 761, *X ^2^* = 1203.12 ( *p* < 0.001).Since chi square goodness of fit tends to be significant when the sample size is greater than 200, we also reported other indices for evaluating model fit. RMSEA was 0.03 (<0.05), CFI was 0.93, and TLI was 0.92 (>0.9), which indicated a good model fit.

**Table 2.** SEM Model Fit Indices

**N of observations**	632
**x ^2^**	1203.12
**d.f.**	761
**p-value**	0.00
**RMSEA**	0.030
**Probability RMSEA <=0.05**	1.000
**CFI**	0.930
**TLI**	0.924

### Measurement Model

Results of confirmatory factor analysis are shown in **Table 3**. All survey items significantly loaded on their corresponding constructs or sub-constructs. Standardized factor loadings, variance explained of each survey item, and the internal consistency (denoted by Cronbach’s alpha) among items measuring the same construct were reported. The majority of the factor loadings were above 0.6 which confirmed the survey’s structural validity ( Tabachnick and Fidell, 2013). For example, *Network of Educators* was strongly correlated with *Community Involvement* ( *b* = 0.91) and *Community Gain* ( *b* = 0.92), and *Education Innovation Project* was strongly correlated with Project Status ( *b* = 0.94) and *Changes by Project* ( *b* = 0.88). The construct *Improved field of HPE* was measured by *HPE Publication* (b= 0.84), *HPE Organization Association* (b = 0.81), and *Conference Organizing/Presenting* (b = 0.71). Cronbach’s alphas ranged from 0.48 to 0.81 for all constructs with the exception of *HPE Publication* (α = 0.19) *.*

**Table 3.** Measurement Model Results

ConstructQuestionnaire Item	Std. Factor Loading (S.E.)	Variance Explained	Cronbach’s Alpha	ConstructQuestionnaire Item	Std. Factor Loading (S.E.)	Variance Explained	Cronbach’s Alpha
**COMM_INV**			**.73**	**COMM_GAIN**			**.75**
CI1	.69 (.04)	.47		CG1	.92 (.03)	.85	
CI2	.82 (.03)	.66		CG2	.87 (.03)	.76	
CI3	.79 (.03)	.62		CG3	.75 (.03)	.56	
CI4	.83 (.03)	.68		CG4	.71 (.03)	.50	
CI5	.73 (.03)	.54		CG5	.67 (.04)	.45	
**KS_APPLY**			**.67**	**PJ_STATUS**			**.48**
KS1	.63 (.05)	.39		PJ1	.43 (.06)	.19	
KS2	.75 (.04)	.56		PJ2	.60 (.06)	.36	
KS3	.61 (.04)	.37		PJ3	.58 (.06)	.34	
KS4	.44 (.05)	.20		PJ4	.59 (.08)	.34	
KS5	.69 (.04)	.47		PJ5	.56 (.12)	.31	
KS6	.74 (.04)	.55		PJ6	.52 (.06)	.27	
**PJ_CHANGE**			**.5** **7**	**COLLAB**			**.62**
PC1	.72 (.05)	.52		RC1	.63 (.05)	.40	
PC2	.63 (.05)	.40		RC2	.71 (.05)	.50	
PC3	.62 (.06)	.38		RC3	.90 (.04)	.81	
PC4	.62 (.06)	.39		RC4	.54 (.09)	.29	
**PUB**			**.19**	**CON**			**.5** **5**
PU1	.25 (.06)	.06		CO1	.71 (.05)	.50	
PU2	.70 (.13)	.49		CO2	.57 (.07)	.32	
				CO3	.76 (.06)	.58	
**HPE_ORG**			**.48**	**HPE_UNIT**			**.48**
HO1	.77 (.06)	.59		HU1	.86 (.09)	.73	
HO2	.82 (.06)	.67		HU2	.67 (.08)	.44	
**HPE_JOUR**			**.58**				
HJ1	.91 (.08)	.83					
HJ2	.84 (.08)	.70					
**Second-order CFA**							
**Network of Educators**			**.81**	**Education Innovation Project**			**.66**
COMM_INV	.91(.05)	.82		PJ_STATUS	.94(.06)	.89	
COMM_GAIN	.92(.05)	.83		PJ_CHANGE	.88(.05)	.78	
**Improving the field of HPE**			**.78**				
KS_APPLY	.26(.08)	.62					
PUB	.84(.16)	.71					
CON	.71(.06)	.51					
HPE_ORG	.81(.06)	.66					
HPE_UNIT	.57(.07)	.32					
HPE_JOUR	.54(.07)	.30					

COMM_INV = Community Involvement, COMM_GAIN = Community Gain, KS_APPLY = Knowledge & Skills Application, PJ_STATUS = Project Status, PJ_CHANGE = Changes by Project, COLLAB = Research Collaboration, PUB = HPE Publication, CON = Conference Organizing/Presenting, HPE_ORG = HPE Organization Association, HPE_UNIT = HPE Unit Association, HPE_JOUR = HPE Journal Association

### Structural Model

**Figure 2.**
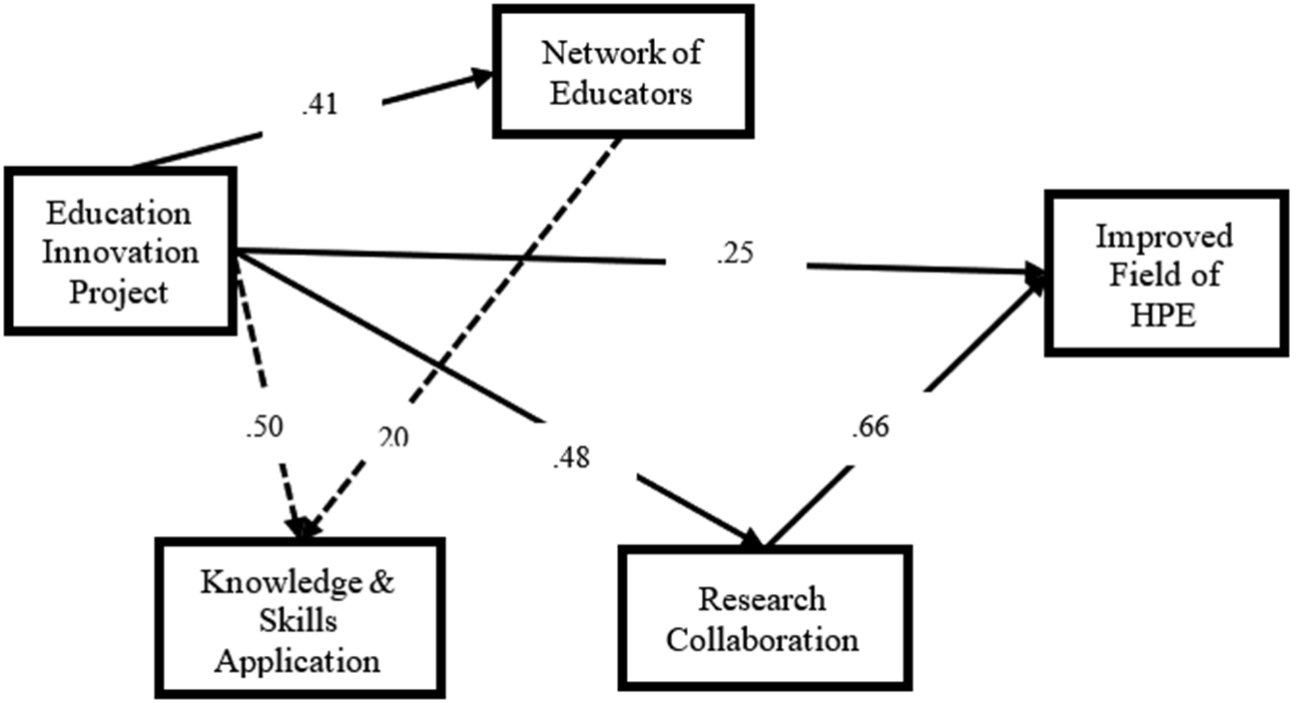
Structural Model Results (revised model with only significant paths p<0.001)
The WLSMV estimation method of Mplus version 7.4 was used. Loadings were standardized. X2 = 1203.12, d.f. = 761, p < 0.001, RMSEA = 0.03, probability (RMSEA <= 0.05) = 1, CFI = 0.93, TLI = 0.924

**Figure 2** shows the result of the structural model. Modification indices output suggested a direct association between *Education Innovation Project* and *Knowledge & Skills Application*, and between *Network of Educators* and *Knowledge & Skills Application.* Overall model fit was improved after adding these two paths to the structural model. Results showed that the construct *Education Innovation Projects* was positively associated with *Network of Educator* ( *b*= 0.41), and *Research Collaboration* was positively associated with *Improved Field of HPE* ( *b* = 0.66) *.* No significant association was found between *Network of Educators* and *Research Collaboration.* Results also indicated that *Education Innovation Project* had a direct positive association with *Improved field of HPE* ( *b* = 0.25), *Research Collaboration* ( *b =* 0.48), and *Knowledge & Skills Application* ( *b = 0.50*). In addition, *Network of Educators* had a direct positive association with *Knowledge & Skills Application* ( *b = 0.20*). No significant association was found between *Network of Educators* and *Improved field of HPE*, or between *Network of Educators* and *Research Collaboration.*

Number indicates the standardized regression coefficient between the measured latent constructs in FAIMER’s theory of change.

Solid line shows paths in theory of change.

Dotted line shows paths not in theory of change but supported by empirical data.

## Discussion

The purpose of this study was to evaluate FAIMER’s fellowship program theory of change by examining the association between latent constructs *Network of Educators*, *Education Innovation Project* and *Research Collaboration*, and their association with *Improved field of HPE.* Results from confirmatory factor analysis showed that the latent constructs were significantly correlated with their corresponding survey items. Second-order confirmatory factor analysis also confirmed the multi-dimensionality of the constructs, *Network of Educators*, *Education Innovation Project* and *Improved field of HPE.* Factor loadings of *Community Involvement* and *Community Gain* on *Network of Educators* seem to suggest that contact between Fellows and exchanges of information were fundamental for enhancement of networks. Factor loadings of the six sub-constructs on the outcome construct *Improved field of HPE* indicate the importance of systems for diffusion of information and career development for health professions educators in improving education.

Results of the structural model show that FAIMER’s impact appeared to have positively influenced advancement of multiple facets of health professions education as anticipated in our theory of change. Several hypothesized pathways in the FAIMER theory of change were supported by empirical data: the direct effect on improving the field of HPE has been supported through increased research collaboration between Fellows and faculty, and implementation of an education innovation project. Project implementation was positively associated with the development of a network of educators. We also found that application of knowledge and skills was directly related to project implementation and network development, pathways that were not depicted in our theory of change. In hindsight, this makes intuitive sense because one of the primary objectives of project implementation is the practical application of knowledge and skills obtained during the fellowship. Our data supports the positive association between *Education Innovation Project* and *Improved Field of HPE.* This would indicate that through institution endorsed education innovation projects, health professions educators from resource constrained settings are applying knowledge and skills to address local needs in health professions education. This may potentially also enhance the role of medical educators as a desirable career goal for faculty in such settings.

Some elements in our model were not supported by the study results, specifically the association between *Network of Educators* and *Research Collaboration.* While strong professional network indicators are generally associated with better research performance ( Abbasi and Altmann, 2011), the association between them could possibly depend on moderating constructs or indirect pathway that are likely missing in the FAIMER’s theory of change. Alternatively, it may not be evident in this sample given the wide geographic spread of the Fellows. Intentional community building is a key feature that contributes to the effectiveness of faculty development ( Steinert *et al.*, 2016), and is also a salient component in FAIMER’s theory of change. FAIMER’s online community provides a supportive learning environment with organized activities to encourage discussion and collaboration between Fellows and faculty, which contributes to the building of a network of educators. As a result of this finding, we may increase the scope of activities related to community building to strengthen the association with research collaboration.

Results of this evaluation study may help improve our program theory. The hypothesized path between *Network of Educators* and *Research Collaboration*, while not supported by analysis of our data, allows us to re-examine the causal relationship between these two constructs and to better explicate the theory. This study will also help us apply a more targeted evaluation approach to conduct in-depth examination of certain constructs in theory of change. Case studies of Fellows’ projects that solicit a wide range of impact inputs may be one method to accomplish this goal. Social network analysis may be another useful adjunct to understand how the network of educators functions and how development of such a network strengthens faculty development ( William P. Burdick, 2014). Better understanding of our program theory may also guide improvements in FAIMER education programs. Our results suggest that scholarship and research collaboration strongly correlate with improving the field of health professions education; therefore we could potentially modify elements of the curriculum to put more weightage on research and scholarships and create more opportunities for Fellows and faculty to collaborate on research. For FAIMER Fellows, a program theory that has been supported by data, may create a clear conceptual pathway for Fellows to improve professionally through implementing their education innovation projects to strengthen knowledge and skills, build network of educators and increase research collaboration. Additionally, since study findings provide empirical evidence that FAIMER’s faculty development program is contributing to improving the field of health professions education, it will help FAIMER to continue seeking program support from stakeholders, such as board members and funders.

The study had a robust sample size. Literature shows that web surveys have a lower response rate than other survey modes ( Manfreda *et al.*, 2008). Baruch and Holtom (2008) reported in their research that the average response rate for email surveys is 54.7%. Many Fellows we surveyed are also physicians, and surveys involving physicians tend to have low response rates ( Guevara *et al.*, 2009; Guevara *et al.*, 2018). This study had a total response rate of 60.2%, and more importantly, most participants completed the survey; the item non-response was 8%.

There were still several limitations present in our study. First, data is self-reported and based on individual reflection. Fellows were asked to report activities during the past one year, which may have resulted in a recall bias in the survey, as respondents may be more likely to report higher levels of participation as the length of recall period increases ( Tarrant *et al.*, 1993). Nonetheless, most of the items for the scales gathered factual information about the participants, which might alleviate social desirability bias. Second, Fellows from recent program years have a larger representation than Fellows from earlier program years in the sample. Similarly, a significant difference in the mean age between respondents and non-respondents was observed, which may have resulted in a response bias in the survey results towards younger Fellows. Non-response may be due to limited availability for those with seniority and leadership roles in institutions, lack of access to the internet, or to low levels of computer skills especially among older age respondents. If we were able to enroll more Fellows in earlier program years in the sample, we anticipate that FAIMER’s impact of improving the field of health professions education would have been stronger.

## Conclusion

Data from Fellows since the inauguration of the FAIMER’s faculty development program provide evidence that FAIMER’s fellowship programs have made an impact of improving the field of health professions education. Using a theoretical framework in evaluation offers us a better understanding of the impact of our intervention and helps us identify areas of outcomes that need to be strengthened for program improvement through data-driven assessment. Since impact data is collected further downstream from faculty development programs, a theory of change that can be measured, enables measurable outcomes to be framed and linked together to provide evidence for the impact of the program. Correlations between nodes in a theoretical framework in evaluation offers a better understanding of why an intervention has a downstream impact and helps identify intermediate outcome domains that need to be strengthened. A theory of change provides a continuous feedback loop to improve the faculty development program and strengthen data supported program evaluation.

## Take Home Messages

• A well-formulated program theory plays a pivotal role in providing a framework for evaluation.
• Program theory is useful in identifying aspects of the program that work or need to be improved.
• Project and research collaboration in faculty development positively influence health professions education.
• Structural equation modelling is useful to analyze linkages between program activities and program goals.

## Notes On Contributors

SHIYAO YUAN, M.S., M.S.Ed. is Evaluation and Data Specialist for Education at FAIMER.

SNIGDHA MUKHERJEE, Ph.D. is Director of Program Evaluation at FAIMER.

RASHMI VYAS, M.D., M.H.P.E. is the Assistant Vice President for Education at FAIMER.

WILLIAM P. BURDICK, M.D., M.S.Ed. is Vice President for Education at FAIMER.

## Declarations

The author has declared that there are no conflicts of interest.

## Ethics Statement

The study protocol was reviewed and approved by University of Pennsylvania Institutional Review Board (IRB), protocol number 812939. All elements of informed consent were included in the information provided to participants. Consent was assumed for all those who chose to participate in the program evaluation without documentation of informed consent. All data were stored and managed with standard guidelines to protect confidentiality.

## External Funding

This article has not had any External Funding
