# Supplementary material for: Using a theory of change for evaluation: has the FAIMER international faculty development program improved the field of health professions education?
Source: MedEdPublish (2016). 2019 Mar 12;8:50. [Version 1] doi: 10.15694/mep.2019.000050.1 (PMC10712606; doi:10.15694/mep.2019.000050.1)
Supplement: Peer review history

# Reviewer report: Reviewer response for version 1

**DOI:** 10.21956/mep.19770.r29694

**Published:** 2019-07-18

**Authors:** Sara Mortaz Hejri

This review has been migrated. The reviewer awarded 4 stars out of 5 As I understand, in this study, the authors developed a questionnaire to evaluate a well-known faculty development program, the FAIMER fellowship. They also used confirmatory factor analysis to validate the questionnaire. Given the fact that a lot of studies, intended to evaluate faculty development (FD) programs, have not used a theoretical framework, the authors should be commended as they have developed their study based on a previously published model. However, I am a little concerned about the generalizability of findings. It seems that the theory used in this study and the data gathered in the study, are limited to the FAIMER fellowship. I am not sure if the framework and the questionnaire could also be used in other FD programs, and if the findings could be applicable to other settings for a wider readership. While I am aware that there are specific features in the FAIMER fellowship (longitudinal program, both online and onsite sessions, project based assignments, …) that distinguish it from a large number of FD programs, I believe there are still a few programs that share those features. This can be further explored by grounding the study on the literature related to theories and frameworks of “capacity building”. Although factor analysis is beyond my area of expertise, to my knowledge, explanatory factor analysis (EFA) seems like a better choice rather than confirmatory factor analysis (CFA). CFA is useful when we have a firm idea about the number of factors based on previous findings of an EFA. EFA aims at exploring variables without a priori fixed number of factors and it can be based only on a general idea or theoretical framework. With this notion, high correlation between some factors that obtained from CFA, might indicate that they are not really separate factors, as it was assumed according the theory, but they could be merged and be considered as a single factor. Applying EFA could have been more helpful because it loads factors based on data. Results of Cronbach’s alpha have not been discussed. This could be important as more than half of the constructs show a less than desirable value. I assume the limited number of items in each factor has resulted in low values, and merging some factors can help with this. Finally, I think framing the manuscript as a validity study for the developed questionnaire could be considered as a suggestion.

Reviewer Expertise:

NA

No decision status is available

# Reviewer report: Reviewer response for version 1

**DOI:** 10.21956/mep.19770.r29696

**Published:** 2019-03-20

**Authors:** Augusto Scalabrini Neto

**Affiliations:** 1 University of Sao Paulo School of Medicine

This review has been migrated. The reviewer awarded 4 stars out of 5 This is a well designed research using program theory and structural equation modelling of the FAIMER programme and the impact of the programme on various elements of a change model.The paper explains very clearly the methodology and limitations, and the text is well written. Conclusions are very clear and robust, and provide new tools to measure faculty development.

Reviewer Expertise:

NA

No decision status is available

# Reviewer report: Reviewer response for version 1

**DOI:** 10.21956/mep.19770.r29695

**Published:** 2019-03-13

**Authors:** Judy McKimm

**Affiliations:** 1 Swansea University

This review has been migrated. The reviewer awarded 4 stars out of 5 This paper provides a robust testing using program theory and structural equation modelling of the FAIMER programme and the impact of the programme on various elements of a change model. The paper is well written and describes the model and processes well, including limitations. I think further research using mixed methods and seeking explanations as to why and how the changes have occurred would help our deeper understanding of the programme and its impact. However, the results provide linkages between different elements of the programme and subsequent change for the participants and it is useful to have more evaluation tools for faculty development tested.

Reviewer Expertise:

NA

No decision status is available
